# Taxonomy of Cooperation and Reciprocity: Beyond Interdisciplinary Social Science Imperialism

**DOI:** 10.1007/s12124-025-09906-7

**Published:** 2025-04-24

**Authors:** Elias L. Khalil

**Affiliations:** School of Economics, Administration and Public Policy, Doha Institute for Graduate Studies, Doha, Qatar

**Keywords:** Cultural Norms (anthropology), Efficiency (economics), Power (political science), Social Roles (sociology), Divided-Psyche (psychology), Beneficence, Quid pro Quo, Intertemporal Allocation, Altruism, Formal Obligations (justice), Informal Obligations (repayment of favors), Gifts, Allegiance, Hegemony, Grants, Philanthropy, Substantive, Integrity, and Transcendental Satisfactions

## Abstract

The literature on cooperation acknowledges different forms of cooperation and their corresponding forms of reciprocity. This paper goes further and shows that most of these different forms are indeed distinct types; hence, the terms “cooperation” and “reciprocity” are portmanteau. This paper proposes a taxonomy of ten types: i) *quid pro quo*; ii) intertemporal allocation; iii) altruism; iv) formal obligations (justice); v) informal obligations (repayment of favors); vi) gifts; vii) allegiance; viii) hegemony; ix) grants; and x) philanthropy. Nonetheless, “beneficence”, defined as the promotion of the good, is common to all ten types. The promotion of the good entails actions that are free from i) opportunism and deception; ii) self-aggrandizement; and iii) malevolence (envy, schadenfreude, etc.). One payoff of the proposed ten-type taxonomy of cooperation/reciprocity is the delineation of five disciplines: anthropology, economics, political science, sociology, and psychology. Each discipline is suitable for the study of one or two types of cooperation/reciprocity. This raises a question: how does each discipline conceive of the other types appropriate for adjacent disciplines? This paper finds that each discipline effectively *sculptures* the other types after its own preconceived mode of conception (toolkit)—amounting to “interdisciplinary social science imperialism.” The proposed ten-type taxonomy promises a transdisciplinary platform that is impartial, i.e., able to help researchers avoid interdisciplinary imperialism. This payoff shows the possibility of unifying the social sciences without interdisciplinary imperialism, i.e., reducing all types of cooperation/reciprocity to one’s favored preconceived toolkit.

## Introduction

This paper undertakes an analytical interrogation of the term “cooperation”. It uncovers ten types of cooperation—a non-exhaustive list—where many of them are barely related to each other:

1. *quid the pro quo*;
2. intertemporal allocation;
3. altruism;
4. formal obligations (justice);
5. informal obligations (repayment of favors);
6. gifts;
7. allegiance;
8. hegemony;
9. grants; and
10. philanthropy.

The proposed ten-type taxonomy of cooperation is based on the delineation of diverse sets of motives/preferences. Each type of cooperation entails a more or less corresponding type of reciprocity. The ten types of cooperation/reciprocity differ, following the differences among the motives/preferences that occasion them.

The term “cooperation”—and its corresponding “reciprocity” phenomenon—is a portmanteau. While there are relationships among some types of cooperation/reciprocity, other types are unrelated (see Table 1--see detailed explanation in the “Conclusion”). Nonetheless, researchers may detect one common feature among the ten types: beneficence. As defined in a standard dictionary, beneficence is “the quality or state of doing or producing good” (Merriam-Webster, 2025). The “good” amounts to what Beauchamp calls acts expressing “mercy, kindness, and charity”:

The term *beneficence* connotes acts of mercy, kindness, and charity and is suggestive of altruism, love, humanity, and promoting the good of others. In ordinary language, the notion is broad, but it is understood more broadly in ethical theory to include effectively all forms of action intended to benefit or promote the good of other people (Beauchamp, 2019).

**Table 1:** Ten-type taxonomy

Type of Beneficent Cooperation/Reciprocity	Genus of Satisfaction	Social Science Discipline	Toolkit (mode of conception
*quid pro quo*	Substantive	Economics	Efficiency
intertemporal allocation	Substantive	Economics	Efficiency
altruism	Substantive	Economics	Efficiency
formal obligations (justice)	Integrity	Political Science	Power
informal obligations (repayment of favors)	Integrity	Sociology	Social Roles
gifts	Transcendental	Anthropology	Cultural Norms
allegiance	Substantive & Transcendental	Anthropology	Cultural Norms
hegemony	Substantive	Political Science	Power
grants	Substantive & Transcendental	Sociology	Social Roles
philanthropy	Substantive & Transcendental	Psychology	Divided-Psyche

This paper goes further and understands “beneficence” more broadly to include all forms of action intended to promote the good of others *as well as the good of the actor*.

Thus, the pursuit of self-interest is beneficial because it promotes the good of the self. What is critical in the definition of the “good” is not acts toward others but rather acts per se insofar as they are grounded in motives/preferences free from at least three intentions:

i) deception and opportunism;
ii) self-aggrandizement; and
iii) malevolence (e.g., envy, rancor, schadenfreude, hate).

This broad definition of “beneficence” overlooks two important differences at the entry-point of analysis. The first is the difference between deception and opportunism. Deception takes place when a decision maker (DM) *ex ante* lies to increase her benefit, which is close to theft and treachery. Opportunism takes place when the DM *ex ante* makes a sincere promise but reneges *ex post* to increase her benefit (Khalil, 2025d).

The second difference, as Ross (2002) argues, is an asymmetry between the prohibition of acts aimed at harming others, on the one hand, and the promotion of acts aimed at enhancing the benefit of others, on the other hand. Ross uses intuition to illustrate this asymmetry. There is an *impending* moral duty to abstain from, say, stealing, whereas there is a non-impending moral duty to make sacrifices to benefit others. The well-known “trolley problem” illustrates the asymmetry, which is useful in medical and legal ethics (Thomson, 1976).^1^

While these two differences are important, they are pertinent for secondary approximation. In the first approximation, the entry-point of this paper, the task is to identify the different types of cooperation/reciprocity broadly conceived.

What is the payoff of the proposed ten-type taxonomy of cooperation/reciprocity? It promises to identify the boundaries among five social science disciplines—namely, anthropology, economics, political science, sociology, and psychology.^2^ Each discipline is ultimately grounded in a preconceived mode of conception, called the “toolkit” for short. The toolkit causes the researchers within the respective discipline to analyze behavior in a particular way—*making the discipline most suited to the study of one or two types of cooperation/reciprocity but less suited to the study of the others*. Most certainly, at the second approximation, each discipline studies all other types of cooperation/reciprocity. However, such interdisciplinary forays are broadly based on each discipline’s preconceived toolkit, leading to misappropriation, if not mutilation, of the other types of cooperation/reciprocity. In other words, interdisciplinary forays have generally led researchers within each discipline to *sculpt other types of cooperation/reciprocity after their own discipline's success with one or two types of cooperation—*i.e.*, after the respective discipline’s preconceived toolkit.*

From the perspective of the philosophy of science, specifically from the standpoint of Ockham’s razor, there is nothing misguiding about having a single toolkit that can easily explain the greatest number of types of cooperation/reciprocity (see Khalil, 1989). What is misguided is to invade other disciplines and misappropriate the types of cooperation/reciprocity that fall under their successful achievement and mold such types after the invader’s toolkit. Boulding (1988, p. viii) called such invasion and misappropriation “imperialism”, signaling the economics discipline as the most notorious in such activity.^3^

However, the other four identified social sciences also exercise their own respective brands of what can generally be called “interdisciplinary social science imperialism.” Salmela and Mäki (2018; see also Mäki, 2020/1) maintain that the emotional reward of belonging and expressing loyalty to one’s peers in the discipline is at least one reason behind interdisciplinary imperialism. While emotional reward may explain the deepening and reinforcement of interdisciplinary imperialism, it cannot explain the origin of the fissures among the diverse toolkits—what this paper aims to do.

The main payoff of the proposed ten-type taxonomy is to expose the differences of the diverse toolkits—hoping to limit the harm of interdisciplinary imperialism. The first step in constructing the taxonomy is to offer an impartial platform recognizing the uniqueness of each toolkit advanced by the respective social science. This impartial platform, called “transdisciplinarity” here, recognizes the varied motives/preferences that each discipline emphasizes—accommodating whatever is proposed by each discipline as the “primary” motive/preferences.

This paper constructs an impartial platform while focusing on one topic—namely, the study of cooperation/reciprocity—to exemplify transdisciplinarity. One can build such impartial platforms while focusing on other topics—such as discrimination, the study of collective action, and ethics-based action. However, given the broad definition of beneficence listed above, the study of cooperation/reciprocity promises to be sufficient enough to allow us to build the most efficient/simplest transdisciplinary platform.

The proposed ten-type taxonomy helps us avoid interdisciplinary imperialism. This effort commences with the DM guided by wide-ranging motives/preferences, giving rise to diverse types of cooperation that can become institutionalized into aggregate phenomena such as markets, civil alliances, social communities ranging from the family, the church, and the firm to political communities ranging from the tribe, the empire, and the modern state. Section 1 presents the approach leading to transdisciplinarity. Section 2 surveys five social science disciplines. Sections 3–12 discuss the ten types of cooperation/reciprocity. Section 13 presents interdisciplinary social science imperialism. Section 14 traces the ten cooperation/reciprocity types to three primordial satisfactions. Section 15 concludes. Appendix 1 briefly presents some implications of the ten-type taxonomy.

## Prelude to Transdisciplinarity

This paper does not start with a sweeping interrogation of the five social science disciplines with respect to cooperation/reciprocity. Rather, it starts with the construction of a transdisciplinary framework of the classification of the different forms of cooperation.

Each discipline largely equips its scientists with its own analytical toolkit. Each discipline-bound toolkit mainly emphasizes one kind of motive/preference. Scientists working within each discipline are enabled to identify a corresponding type of cooperation/reciprocity. Consequently, such scientists tend to study the other kinds of motives/preferences emphasized by adjacent disciplines in secondary, if not tertiary, approximations.

Stated simply, the preconceived toolkit of scientists working in each discipline tends to direct them to view all motives/preferences and, corollary, all types of cooperation through a limited set of lenses. They are limited in the sense that they are suitable for one or two types of cooperation.

This statement should not mean that scientists in one discipline ignore the motives emphasized by other disciplines. These scientists usually recognize all other motives discussed by other disciplines. However, they either relegate such motives to a secondary, if not tertiary, status or, more ambitiously, they reduce them to the primordial motive that characterizes their own discipline-bound toolkit.

Furthermore, this statement should not mean that each discipline is monolithic. Each discipline rather consists of diverse schools and perspectives that are engaged in debates of various degrees of intensity at different periods in the life of the discipline. There are a few anthropologists who have advanced the economist approach to the study of culture (e.g., Harris, 2001). Moreover, few economists are comfortable with the anthropological approach to the study of markets and organizations (e.g., Veblen, 1899). Indeed, approaches in (heterodox) economics consider *power*, the toolkit of political science, to trump efficiency, the toolkit of standard economics: feminist economists highlight power along gender categories (e.g., Ferber & Nelson, 1993); radical economists stress power along class categories (e.g., Marglin, 1974); institutional economists emphasize power along market monopoly categories (e.g., Galbraith, 1985); and inequality economists focus on power along racial and ethnic categories (e.g., Hardy & Logan, 2020).

Furthermore, a few sociologists subscribe to the economist rational choice model in understanding individual choices and the behavior of corporate actors (organizations) (e.g., Coleman, 1994). Moreover, some economists advance the sociologist emphasis on social roles, peer pressure, and the quest after status (e.g., Frank, 1985, 2000). In addition, a few psychologists ultimately resort to the neoclassical economist’s sense of bounded rationality (understood as being based on cognitive economy) to explain heuristics and rules-of-thumb (e.g., Kahneman, 2011; see Khalil, 2022; Khalil & Amin, 2023). Moreover, there are economists who are committed to the psychologist focus on whimsical emotions, visceral facts, and the importance of context (e.g., Loewenstein, 2007).

Nevertheless, while social science disciplines are non-monolithic, there are ultimately clear conceptual boundaries that divide the social science disciplines. The disciplines do not exchange ideas in an open-air bazaar that allows researchers, say, in sociology, to easily adopt concepts from economics or psychology. While researchers in each discipline might be ready to listen to imported concepts from other disciplines, discussions within a discipline are ultimately regulated by the commitment of the discipline to a core mode of conception. In the final analysis, discussions within the discipline remain local and are restricted by the dominant core toolkit that typifies the discipline.

Thus, at first approximation, we should be permitted to identify the conceptual core of each discipline, which is called below the “typical” or “stylized” representation of the discipline. Only at the second approximation can we acknowledge that not all scientists within the respective discipline subscribe to its typical core.

## Five Social Science Disciplines: Five Toolkits

### The Stylized Anthropologist: Cultural Norms

To start with the stylized (i.e., typical) anthropologist, she usually stresses the function of *cultural norms* as invisible threads that constitute the communal fabric. For example, Douglas (1986) highlights how categories and concepts that individuals use are not created by those individuals but rather are the products of the invisible institutions that sustain the community. In addition, Douglas and Isherwood (1996) argue that the material goods that people consume do not merely satisfy substantive needs. They also satisfy the need to communicate with each other, assisting in strengthening bonding relationships, identity formations, and social stratifications.

Furthermore, Bateson (1935) and later Graeber and Wengrow (2021) reason that the primary quest of communities is to signal their unique identity: communities use goods as signals to differentiate themselves from nearby communities. In the process, the quest after identity ensues dynamics, what Bateson calls “schismogenesis”, which sets communities apart from each other (see Khalil, 2023, 2025c).

The ultimate toolkit of stylized anthropologists is culture-based institutions that can be called “cultural norms”—despite the protest of anthropologists such as Geertz (1977) regarding the overuse of the term “culture”. The centrality of “cultural norms” as a conceptual toolkit is most suited to one type of cooperation/reciprocity: what is called below “beneficence-as-gift.” Gifts are central in sustaining the fabric of families, neighborhoods, and communal associations. As the term used here, “gifts” are limited primarily to the symbolic function of expressing love. There are other carriers of love, discussed below as “grants” and “philanthropy”, but such carriers amalgamate love with a substantive function, i.e., in sustaining material life in a serious manner. The distinction of gifts, on the one hand, and grants and philanthropy, on the other hand, is not solid, as gifts usually have substantive elements (see Khalil, 2024a).

As for symbolic function, it appears as the set of rituals employed to reproduce identity, facilitate bonding with family members, and even build friendships and fondness beyond kinship relationships (Smith, 1976a, pp. 220–222).

In addition, “cultural norms” as a conceptual toolkit are useful in the analysis of another type of cooperation/reciprocity: what is called “beneficence-as-allegiance.” A DM expresses allegiance when she submits to the authority of a parent, employer, chieftain, political leader, or the state. Such submission involves giving up some parts of one’s autonomy in a nontrivial sense. That is, allegiance-based submission goes beyond trivial abidance to dress codes, traffic laws, and measurement standards such as the metric system.

What does submission mean in nontrivial sense? This paper relies on the neglected work of an anthropologist, Steiner (1949), in characterizing allegiance-based submissions. Allegiance involves submission that differs from surrendering to the hegemon. Allegiance is submission to a parent/employer/leader who takes the function of “guardianship.” As a guardian, the parent/employer/leader is fully committed to guarding the best interest of the subject. In contrast, the hegemon is not.

### The Stylized Economist: Efficiency

There is a misperception of the toolkit of the stylized economist that is common not only to the wider public but also to most social scientists, including many economists such as one of the founders of modern (neoclassical) economics, Alfred Marshall, 1920; Khalil, 1995c, 1996b. Specifically, it is the claim that economics is about the study of the pursuits of material comfort, where decision makers (DMs) act rationally and motivated by self-interest.

It is true that standard economists construct models of rational DMs motivated to maximize objective functions consisting of bundles of goods that promote their own benefit. However, as Becker (1974) and long before him, Robbins (2007), in his classic 1932 book, emphasized, this common view of the toolkit of economics is only partially true. It is true that choices are the outcome of maximization, i.e., the outcome of rational decisions. Rational decisions, defined briefly, arise from coherent motives/preferences, whereas the DM does not pay attention to promises or commitments made in the past. The DM ultimately attends forward-looking cost‒benefit calculations—while considering the preferences, resources, and available technological–institutional regimes as given.

What is wrong about the common view of the toolkit of economics is the supposition that the objective function of DMs consists only of bundles of goods that promote their own benefit. This is not the case. The objective function may include bundles of goods that promote the benefit of other people. As such, the toolkit of economics is *efficiency*: what is the most efficient means to maximize the benefits of whomever included in the objective function? As the toolkit specifies, the DM must search and buy the best bundle of flowers and chocolate when visiting a friend in the hospital or the DM must decide how often to visit the friend as Hahn (1991, p. 8) quips. The economist toolkit does not specify that the DM has only self-regarding preferences.

For the economist toolkit, the DM can be equally motivated to help needy others (altruism); to save the planet from pollution, consumerism, racism, sexism, war, and diseases (public spiritedness); to offer care to children and family members (love); to purse what affords pride and dignity (aspiration); and even to enjoy envy of the fortunate and schadenfreude of the unfortunate (malevolence). In his 1932 book, Robbins emphatically postulates that the toolkit of economics is about economizing—choosing the most efficient means to satisfy preferences where such preferences are not limited to self-interest. Becker (1993) affirms this postulate—what philosophers call “instrumental rationality” (e.g., Kolodny & Brunero, 2023).

The efficiency toolkit is well suited for the analysis of one type of cooperation/reciprocity: what is called “beneficence-as-altruism.” When the DM acts altruistically, she wants to promote other interests—but in an efficient manner. As Becker (1976) shows, the altruist’s act is efficient. The altruist has to contrast her own marginal cost vis-à-vis the marginal benefit of the beneficiary—while taking her altruist preferences toward the beneficiary as given. Thus, the altruist would help when it is obvious that the other would benefit from one unit of assistance more than she would. In addition, the altruist tends to help more familiar people than nonfamiliar others, given their altruistic preferences (see also Khalil, 2009).

We should not fail to mention the iconic function of the economist toolkit: the analysis of decisions taken to promote self-interest. The pursuit of self-interest reflects two types of cooperation/reciprocity. The first type of self-interest is obvious transactions, called “beneficence-as-*quid-pro-quo*”, where two or more DMs meet and make a contract to exchange goods. In modern society, the market has become the dominant institution facilitating *quid-pro quo* transactions. However, other institutions can equally take place within organizations to facilitate *quid pro quo* transactions, such as intra-family, intra-firm, and intra-government bargaining.

The second type of self-interest, which is less obvious, is investment/saving activity. It is less obvious because it requires us to conceive the DM as a composite of at least two selves: the present self and the future self (Elster, 1986). When the present self invests/saves resources for future consumption, it is an act of current sacrifice to enhance the wellbeing of the future self. The act of saving/investing, hence, is an act of cooperation. This form of cooperation involves intertemporal allocation: what is called “beneficence-as-intertemporal-allocation.”

### The Stylized Political Scientist: Power

While the toolkit of the economist is efficiency, the toolkit of the stylized political scientist is *power*. The quest after power differs from the quest after wealth or the efficient pursuit of wealth. While power and wealth usually support each other, the (efficient) quest after wealth for the political scientist is primarily a means to harness power rather than vice versa.

The stylized political scientist’s entry-point, i.e., power, differs from Hobbes’ (2017)—even though Hobbes’ social contract theory is an attempt to explain the emergence of the power of the state. Hobbes and other advocates of social contract theory reason that DMs, when they confer power to the sovereign, they do so to maximize self-interest. Otherwise, there is no law and order, which is needed to avoid slipping into the anarchy of the state of nature. Thus, Hobbes and other social contract theorists reason that the state is a tool designed to enhance wellbeing through the suppression of anarchy, where anarchy usually, but not necessarily, invites suboptimal subgame perfect Nash equilibrium.

The basis of the toolkit of stylized political scientists is Machiavelli’s (1992) *The Prince*. Aside from the wide scholarship and multitude of interpretations of Machiavelli (e.g., Mansfield, 1998), the core idea is how the prince, who is interested in power, should manipulate his subordinates, confuse his allies, and even spread rumors and lies to sustain and increase his grip over power. As such, the most suitable type of cooperation/reciprocity for stylized political scientists is “beneficence-as-hegemony”—the dedicated effort of the DM to amass asymmetric power to attain hegemony over the will of others.

Another suitable type of cooperation/reciprocity is what is called “beneficence-as-formal-obligation.” This type is grounded in the reciprocal recognition of rights, i.e., the principles of justice. The pursuit of justice, however, need not be prompted by power. It can, as noted above in reference to Hobbes and other advocates of social contract theory, be prompted by substantive self-interest. However, political scientists are interested in beneficence-as-formal-obligation as it occasions, upon acting according to the principles of justice, a kind of satisfaction, called “integrity,” that is incommensurable with the substantive satisfaction arising from the consumption of everyday, substantive goods such as food, shelter, and entertainment.

### The Stylized Sociologist: Social Roles

The typical sociologist, following the lead of Durkheim (1982), regards the pursuit of wellbeing or interest to be the fulfillment of one’s role or function in organizations and society at large. Thus, the toolkit *social roles* captures for stylized sociologists the entry-point of understanding the actions of DMs.

The actions that individuals undertake are principally molded by existing social attitudes and norms. Individuals seek the approval of other people in their respective social network. Hence, they eagerly perform acts that validate “social facts,”, i.e., the expectations of other people who make up one’s social milieu. This process amounts to the internalization of social norms. George Herbert Mead (2015) perfected sociological theory, providing a detailed micro-level, psychological mechanism behind the internalization of social norms.

Given that the toolkit of sociology is the socialization of the individual according to more-or-less given “social roles”, it is well suited to the analysis of one type of cooperation/reciprocity: what is called below “beneficence-as-repayment.” Even if there is little expected punishment in terms of legal sanctions, the DM feels obligated to payback favors in order to be accepted, favored, or praised by others. This feeling amounts to the internalization of duty, which is the foundation of social capital (see Coleman, 1994; Putnam, 2020).

This characterization of the toolkit of sociology is somewhat limited to classical sociology, taking its form in Durkheim’s structural–functional approach. Contemporary sociological theory, e.g., that of Giddens (1979, 1984) and Bourdieu and Wacquant (1992), came to acknowledge the role of individual autonomy or agency: the action of the individual does not express only the internalization of social roles. The action also externalizes the inner conflicting desires of the DM, which, through action, are responsible for the production of social structures. Contemporary sociological theory basically attempts to reconcile these two poles: the primacy of the “structure” of society in explaining individual action, on the one hand, and the importance of the “agency” of the autonomous individual in producing the structure of society, on the other hand. This has resulted in varied and rich literature with contested ideas, known as the structure‒agent debate (see Ritzer, 2000).

While contemporary sociological theory pays close attention to individual autonomy, individual desires, and personal quest for emancipation, it broadly does not offer a systematic account of human action. Specifically, it does not offer a theory of how to relate the diverse motives/preferences to each other in its account of action. The focus on individual autonomy and desires produces, in the final analysis, secondary qualifications of the classical toolkit. Thus, at least for the purpose of this paper, we may consider the classical sociological toolkit, i.e., social roles, to still express the stylized sociologist approach.

### The Stylized Psychologist: Divided-Psyche

It is difficult to identify a single toolkit that typifies psychology—as the approaches from psychodynamics (Freud), behaviorism (Pavlov and Skinner), cognitivism, and cultural, evolutionary, biological, and humanistic psychology (Rogers and Maslow) are far apart. However, one unifying theme across these approaches is the view of the psyche as a conflicting entity, ridden with anxiety, and beset of upheavals of emotions. The self is essentially not composed of coherent motives/preferences, contrary to the supposition of standard rational choice theory. As a result of anxiety, the self is rather split as if the DM lives with two minds, conceiving the world in conflicting ways, which Laing (1965) sums up as the “divided self”.

The notion of the “divided self” goes back to Plato (Barney et al., 2012), infuses Sufi Islamic philosophy (e.g., Al-Ghazali, 2000) and permeates existentialist Christian philosophy (e.g., Kierkegaard, 1981, 1986). It also suffuses existentialist themes in the modern novels of Dostoevsky and Kafka (see Hubben, 1997). However, there are many notions of the divided self—such as the present self vs. the future self and the hedonistic self vs. the transcendental self. To avoid the diverse connotations of the term, this paper instead uses the term “*divided-psyche*” to denote the overarching toolkit.

The stylistic psychologist studies behavior as stemming from the everlasting struggle of the DM to reconcile the different, and sometimes conflicting, parts of the psyche. The stylistic psychologist tends to view behavior as whimsical, where the DM is pulled in different directions with no steady purpose or motive. The motives/preferences are basically incoherent or at least foamy in the sense of indeterminate basically because of anxiety—where the different approaches trace anxiety to different sources ranging from unconscious desires (psychodynamics), neural functions (biological), the environment (behaviorism), cultural norms, decision making (cognitivism), and evolution to the humanistic perspective of needs.

With respect to foamy motives/preferences, the cliché examples include consumer behavior. The consumer is ready to buy one brand rather than another simply because of some hidden clues, such as status appeal or sexual allure. Alternatively, the consumer may change his or her choice simply because of what behavioral scientists call the “primacy effect” (see Dhami, 2017, 2025). This effect amounts to choices that depend on heuristics that the DM follows depending on first encounters or first impressions (Sullivan, 2019).

Furthermore, facts and information about the environment seem to have little effect on one’s already held beliefs. In experiments, DMs seem to focus more on evidence that supports their prior beliefs and overlook evidence that challenges them—what psychologists call “attention bias” (e.g., Kuhn et al., 1988)). When experimenters present useless information, DMs still interpret it as supportive of their prior beliefs (e.g., Kuhn, 1991).

However, the concept “divided-psyche” is more common than foamy preferences and fact-free beliefs. The DM can be the subject of upheavals of emotions resulting from deep anxieties. Setting aside the many criticisms of Freud (e.g., Levy, 1996; Robinson, 1993), he postulated that most modern pathologies arise from anxieties to be traced to the failure of the DM to reconcile a divided psyche: while one conscious psyche (the “superego”) expresses proper and moral choices, another subconscious psyche (the “id”) expresses primeval impulses and desires (Freud, 1949, 2010a; see Sarno, 2007). Indeed, Freud (2010b) regarded civilization as the playground of the inescapable tension arising from such divided psyche.

Despite the widespread dismissal of Freud’s basic concepts, stylized psychologists still have to address the inconsistent and varied behavior stemming from the divided psyche. They do not need to rely on Freud’s basic concepts to investigate the self’s fissures that become apparent in the analysis of beliefs DMs produce to justify, e.g., actions. The DMs resort to self-deception, delusions, and other forms of distorted beliefs (Khalil, 2016, 2017a). The self’s fissures also become apparent in the analysis of beliefs produced to justify the violations of moral principles. As the concept of cognitive dissonance illustrates, people invent justifications when their actions deviate from their beliefs regarding moral principles (Festinger, 1957).

Haidt (2001) even goes further. DMs may not even start with beliefs regarding moral principles but rather with emotions. DMs simply have repulsions toward certain acts—and later, they appeal to moral principles to rationalize such emotions. That is, DMs seek justifications after they experience emotional repulsion or moral outrage. To appear as a person with autonomous agency, the decision maker uses rational justification only ex post. What motivate actions are rather accidental encounters and emotional upheavals.

Greene (2014) shows how moral sentiments are not derived from some axiomatic fundamentals. Such sentiments serve two purposes: they make us suitable to our society while at the same time informing social identities that separate us from other communities. For Greene, such sentiments are the raw materials produced by evolutionary processes.

In this regard, the primary toolkit of psychologists, or at least the views of Haidt and Greene, can largely be traced to David Hume: “Reason is, and ought only to be the slave of the passions” (Hume, 2007: 2.3.3). This statement, as Sugden (2008) convincingly clarifies, is not about the centrality of rational choice, i.e., affirming that reason is the instrument used by people to maximize preferences (passions). It rather expresses the gist of Hume’s theory of human nature: Reason has no authority in either science or morality; it is the passions that have such authority. That is, passions trump reason. Within the context of his discussion of moral psychology, Hume postulates that passions, including moral inclinations and emotional upheavals, cannot be explained or justified by reason. The passions are the entry-point of understanding behavior, whereas reason arrives at the scene late, only to justify the emotion-driven actions.

On the basis of Sugden’s clarification, Hume should be the father of modern psychology. Like no other modern thinker, Hume has squarely postulated that the divided-psyche, given its upheavals and divisions, is the entry-point for understanding human behavior.^4^

The divided-psyche toolkit of the stylized psychologist is suitable for uncovering one type of cooperation/reciprocity: what is called “beneficence-as-philanthropy.” A DM undertakes acts of philanthropy--ranging from leaving enormous wealth to children beyond duty, hospitals, hometowns, and universities--for the expressed purpose, ignoring the literal translation of “philanthropy”, to glorify one’s legacy. Such acts can be understood only as examples of divided-psyche: the DM expands the self beyond the living self to abridge a divided self. The DM undertakes philanthropic actions to allow the living self to live beyond the grave, i.e., to exist beyond the biological skin of the living self. The concern with one’s legacy beyond the grave is a well-recognized motive—and even exalted and cherished across cultures.

### Recapitulate

This paper presents five social science disciplines and their corresponding five toolkits. Each toolkit is proven to be suitable for one or two types of cooperation/reciprocity—but no single toolkit is suitable for all ten types. By distinguishing toolkits and showing their limited scope, this paper provides an impartial (transdisciplinary) platform from which researchers can respect the boundary and toolkit of each social discipline. Such transdisciplinary platform would make us aware of interdisciplinary social science imperialism.

This transdisciplinary platform allows us to capture the different forms of beneficence: how cooperation/reciprocity consists of many types. Although the ten identified types are beneficial, they inform different kinds of choices or behaviors, as shown next.

## Beneficence-as-*Quid-Pro-Quo*

The most ubiquitous medium of cooperation/reciprocity in modern societies is market-mediated transactions. Such transactions are based on the reciprocal recognition of rights and, hence, can ultimately be grounded in *quid pro quo.* As such, beneficence-as-*quid pro quo* is not the product of modern societies. Such transactions exist in the pre-market setting in all societies and even take place within families, firms, and other organizations, where the price is not determined by the free and competitive market but by relative bargaining power. Even in a market characterized by asymmetric power, where the price deviates from the competitive or “just price”, the transaction is still based on the *quid pro quo* context.

Smith’s expression of the *quid pro quo* context, where each trader appeals to the interest of the other, i.e., does not appeal to the benevolence or humanity of the other, is pertinent:

It is not from the benevolence of the butcher, the brewer, or the baker, that we expect our dinner, but from their regard to their own interest. We address ourselves, not to their humanity but to their self-love, and never talk to them of our own necessities but of their advantages. Nobody but a beggar chuses to depend chiefly upon the benevolence of his fellow-citizens (Smith, 1976b, pp. 26–27).

As argued elsewhere (Khalil & Marciano, 2021a, 2021b), Smith exaggerated his celebration of “own interest” when he characterized the dependence on “the benevolence of his fellow-citizens” as panhandling.

In any case, Smith not only identified market-mediated exchanges as being based on “self-love” and “own interest” but also acknowledged that such motives are demanded by nature (Smith, 1976a, p. 219). He qualified his celebration of the self-interest motive, however, by noting that it must respect the principles of justice. That is, traders are justified in pursuing their own benefit as long as they abstain from deception, cheating, and treachery (Smith, 1976a, pp. 78–91).

## Beneficence-as-Intertemporal-Allocation

When we mention the concept of “self-interest”, we only occasionally note the difference between the present self and future self. When the DM makes a choice, she must evaluate how satisfying the current self may negatively impact the resources available for the future self, what economists call “intertemporal allocation.” For example, if the DM spends all the resources on vacations and leisure, her future self will most likely suffer. Hence, intertemporal allocation is nothing other than the DM balancing between the interest of the present self and the interest of the future self, i.e., a form of cooperation (e.g., Elster, 1985, 1986). Such cooperation entails beneficence, where the DM is standing in the present but with the benevolent aim of making decisions that positively impact the wellbeing of the future self. Hence, we can conceive addiction and slipping into hate and other malevolent acts as acts that are contrary to beneficence insofar as such acts impair the ability of the future self to sustain itself.

Thus, the pursuit of one’s own benefit in a non-myopic way is an act of cooperation. Parents, educators, and caring others level praise at individuals who exercise, eat healthy, avoid under-saving, and invest in human capital. All these acts of cooperation show that the present self has beneficial intentions toward the future self. Indeed, parents, educators and so on even level praise at the DM when the DM takes care of the present self—such as avoiding reckless endeavors or enjoying leisure after a hard working day. Smith (1976a, 1976b) actually regarded the pursuit of self-interest (as long as it does not violate the interest of others) as a virtue, what nature recommends:

EVERY man, as the Stoics used to say, is first and principally recommended to his own care; and every man is certainly, in every respect, fitter and abler to take care of himself than of any other person. Every man feels his own pleasures and his own pains more sensibly than those of other people. The former are the original sensations; the latter the reflected or sympathetic images of those sensations. The former may be said to be the substance; the latter the shadow.

After himself, the members of his own family, those who usually live in the same house with him, his parents, his children, his brothers and sisters, are naturally the objects of his warmest affections (Smith, 1976a, p. 219).

Thus, when the DM takes care of the future self, the DM must, at least equally, be virtuous, what Smith calls “prudence.”

Beneficence-as-intertemporal-allocation is the most elemental form of cooperation. As Kavka (1993) argues, problems that beset cooperation in public goods games among DMs also beset intra-personal cooperation, i.e., how the present self cooperates with the future self. However, how can researchers conceive of intertemporal allocation as intra–personal cooperation when the future self cannot reciprocate, i.e., cannot reward the present self for its sacrifices? Alternatively, how could researchers view it as intra-personal cooperation when the future self cannot punish the present self for discounting the interest of the future self?

Stated differently, it is easy to see cooperation in the market or even in public good games, where the parties obey written or implicit property rights, i.e., comply with the formal and informal norms of justice. As shown below, if the DM cheats others, others can retaliate, i.e., punish the cheater. They can do so via the judicial system or via social norms that scold the cheater. Thus, the non-myopic DM refrains from cheating if the punishment is greater than the expected gains from cheating. If the cheater is allowed to prosper, i.e., without any form of legal or social sanctions, others will also cheat, leading to a suboptimal Pareto outcome for all. In contrast, the future self evidently cannot retaliate and punish the cheater, the present self. Thus, it appears that we cannot model beneficence-as-intertemporal-allocation à la cooperation that typifies public good games or market exchange in general.

However, as stated in detail elsewhere (see Khalil, 2020), the future self can indeed retaliate and impact the wellbeing of the present self. If the present self employs defection strategies, i.e., cheating the future self, the future self can resort to legal and social sanctions via two mechanisms. The parent of the cheater is prompted to withdraw an inheritance as punishment of the choices of the offspring, who is harming her future self. Why should the parent provide support to offspring whose objective function includes only current pleasures? The parent can reason, “I deserve these resources better than my spoiled son; I better spend them on my own current pleasures than offering them to another person to enjoy them.”

After the parent dies, the offspring who inherits the resources can cheat and leave nothing for her own offspring. The long arm of the future self, i.e., the parent, can no longer punish the cheating offspring. However, the same situation occurs in cooperation among contemporaneous DMs. A person can trust and invest resources in the venture proposed by a friend, and the friend can flee the scene with the resources. The fact that a son can also consume all the resources, leaving nothing to the future self or his children, does not invalidate the analysis that intertemporal allocation is similar to cooperation among contemporaneous DMs.

In more complex modern societies, the state takes the function of the parent in this regard. It enacts diverse programs to encourage offspring to undertake savings and other sacrifices that benefit the future self. For example, the state offers subsidies to students to invest in their human capital—and stops such subsidies if the students skip school. Such subsidies are investments amounting to incentivizing the present self to undertake sacrifices to improve the wellbeing of the future self.

Another example is that the state offers subsidies to parents who decide to have children, whose cost of rearing can be seen as sacrifices incurred by the present self to help the future self taking the form of offspring. Likewise, the state provides, on many occasions, subsidies to finance first-home buyers—which is another form of enhancing the present self to undertake sacrifices.

Insofar as members of an economic society promote social norms and legal sanctions that punish cheaters in public goods games, they also promote similar norms and legal sanctions that suppress intra-personal cheating. Thus, at least analytically speaking, we can conceive of beneficence-as-intertemporal allocation—viz., where the DM’s present self assists the future self—to be an elemental form of cooperation and reciprocity.

## Beneficence-as-Altruism

The DM acts beneficently in the sense of altruism when she helps another person in need. The DM is ready to help others who, say, are involved in an automobile accident or whose home has become submerged as a result of floods. When the DM rushes and offers help, she does not expect any payback or award. Otherwise, the act would be based on self-interest, not other-interest.

There are diverse theories of altruism understood as helping others without the expectation of payback. One theory that is current in the economics literature can be traced to the ego-centered theory of Becker (1976). He argues that altruism is the pursuit of self-interest via other means. Rather than directly enjoying, say, warm clothes, the DM is ready to give away clothes to a needy other insofar as the DM can vicariously enjoy the utility of the other. As Hobbes explains, the altruist is not ultimately interested in the wellbeing of the other but rather in her own vicarious enjoyment in witnessing the enjoyment of the other (see Khalil, 2004).

There are other variables that are relevant in such a theory, such as the degree of familiarity. Indeed, this variable is relevant in the highly similar “inclusive fitness” hypothesis, which Hamilton (1964) advances to explain altruism among organisms per se. The hypothesis supposes that an organism is ready to help others if the others share at least some of its genetic make-up—where such sharing is analytically identical to the familiarity notion in ego-centered theory in the economics literature (see Khalil, 2009). For the inclusive fitness hypothesis, when an organism helps a kin, it actually helps the propagation of its own genetic make-up.

Even within the ego-centered conception of altruism à la standard economics or à la evolutionary biology, there is no need for the recipient to reciprocate. As stated above, if reciprocation is part of the calculus of offering help, the offering is no longer an altruism-based action. The offering would be motivated by self-interest—a motive that explains the origin of rules of fairness or justice, as shown next.

## Beneficence-as-Formal-Obligation (Justice)

In free and competitive market-mediated transactions, the parties can pursue their own goals as long as they respect the rights of others, i.e., obey the principles of justice. If there is coercion, theft, or deception, the exchange is no longer regulated by the reciprocal respect of each other’s rights. Even when the exchange is *quid pro quo*—i.e., is not mediated by market-determined prices but by the bargaining of two parties—each party must observe the rights of the other as a prerequisite condition.

The bargaining outcome becomes complicated if the two parties do not have symmetrical power. However, as long as it is free, the cooperation that the two parties enter can be reasoned to stand on the formal obligation of the reciprocal recognition of rights, the basis of justice.

When the DM insists that others should respect her rights, she is ready, although within limits, to incur costs to punish those who violate her rights. The famous ultimatum game illustrates such self-inflicted costs (Güth et al., 1982, 1996). Sen (1977, p. 328) calls the readiness to incur costs to uphold principles, e.g., the equity principle in the ultimatum game or honoring formal obligations entered via contracts, as involving, in a very real sense, “counterpreferential choice”. For Sen, such a cost expresses the formal obligation of the DM, stemming from adherence to the principle of choosing what is the right thing to do. Sen went further and criticized the toolkit of the stylized economist of lacking the ability to capture principled, counterpreerential choice. In Sen’s term, the economist toolkit portrays DMs as “rational fools”—acting as if principles of justice do not matter (Khalil, 1999; see also Sen, 1997).

Not all principles of justice, i.e., formal obligations, are written into law. Only a subset of formal obligations is transformed into grounds for legal litigation. The reason is simple. It is usually expensive to litigate all disputes in court. For example, making noise at a public park usually violates a formal obligation—as it infringes on the rights of others to enjoy the quietness of the park. However, the violation of the formal obligation may not be a violation of a law, as the formal obligation may not have yet been written into a code of law.

Regardless of whether formal obligations are written into a code of law, the evidence afforded by behavioral economics experiments involving the ultimatum and similar other games confirms the readiness of people to inflict costly punishments on violators of formal obligations toward the self—even when there is no benefit to the self (e.g., Henrich et al., 2005; Güth & Kocher, 2015; Dhami, 2025, pp. 152–155). More remarkably, the evidence also confirms the readiness of people as third parties to inflict costly punishment—i.e., when they are bystanders who punish transgressors for inflicting injury on others (e.g., Fehr & Fischbacher, 2004). When people enforce property rights with no self-regarding benefit, they must be motivated by deep moral sentiment, such as the resentment of injustice. For such resentment to be justified, transgressors must have violated an agreement even when it is tacit or entailed by principles of justice commonly held in society.

If a researcher starts with the moral sentiments associated with the quest after justice, say, the resentment of injustice, to explain the rules of justice—as opposed to the contrary—a problem arises. What is the scope of justice? To avoid this dilemma, we need to start with agreements among DMs as the foundation of rights—where moral resentment of injustice can arise only in light of such agreements. This is what Hume (2007) has argued: justice cannot be a “natural virtue”—it is rather derived from self-interest and circumstances. Therefore, he regarded justice as an “artificial virtue” (Hume, 2007, 3.2.1.1; see Khalil, 2025g).

Hume’s view of justice has immediate ramifications with respect to the debate of whether the much powerful Europeans should observe the supposed rights of the indigenous tribes of the Americas. As detailed elsewhere (Khalil, 2013), Europeans are not obligated to honor the property of indigenous Americans, as for Hume justice is ultimately founded on self-interest. The DM obeys the rights of others to secure their own respect for her rights; hence, both can benefit from avoiding the prisoner’s dilemma outcome.

In contrast, for Smith (1976a, pp. 136–137), Europeans have an obligation to honor the property of weaker Americans, beyond the mere beneficence-as-altruism arising from sympathy (see Khalil, 2017b). In Smith’s discussion of the death of millions in an earthquake in China, the great call to observe the property of unfamiliar Chinese (and likewise, weaker Native Americans) stems from a commitment to principles that bind all humanity, the love of other humans, simply because we all belong to the same family.

As detailed elsewhere (Khalil, 2013), Smith’s position is problematic. What determines the degree of commitment to the rights of all members of a species? Why is the *Homo sapiens* species a privileged taxon? Why not the apes taxon? Or why not a lower taxon such as membership in a civilizational cultural group?

In other words, why would DMs consent to rules under which they bind themselves? They must be motivated by self-preservation, as Hobbes supposes, or the establishment of institutions that help them avoid prisoner’s dilemma outcomes, as modern economists argue.

With respect to the deep feeling of resentment, when transgressors injure the integrity of the DM under focus, as discussed elsewhere (Khalil, 2024b), while it is true that integrity and the feeling of resentment are incongruent with substantive utility, we should not conceive the quest after justice as a stand-alone preference. The quest and demand for integrity are products of already existing agreements, which are usually based on the protection and maximization of substantive utility, i.e., wellbeing or interest.

To sum up, contrary to Sen (1977; Khalil, 1999), while standing with justice gives rise to the beneficial feeling of integrity, this feeling is a by-product of what is already agreed upon as a convention à la Hume. The DM is ready to restrict own action insofar as others abide by the agreement. Thus, by definition, justice involves reciprocity: one is ready to respect the rights of others if they observe the original contract.

## Beneficence-as-Informal-Obligation (Repayment)

The DM acts beneficently when he fulfills another kind of obligation, namely, an informal obligation. Informal obligations include the repayment of favors, e.g., when a car mechanic patronizes a restaurant whose owner is his client. The mechanic is not obligated formally to patronize the restaurant but feels that he is obligated somehow. In another example, the DM hires a familiar plumber to perform repairs as repayment for the free extra work that the plumber has rendered in a previous task. This extra work was clearly done to win the return of the customer in a second round. There is nothing deceptive in such calculated self-interest on the part of the plumber, i.e., rendering free extra work in order to be hired again. The restaurant owner and the plumber can be explicit, stating that they hire the mechanic or offer free extra work to win their patronage in the future (Khalil, 2025f).

That is, the tit-for-tat responses or repayment of favors do not amount to “using” people, i.e., acting opportunistically, as long as the initial provider of the favor is sincere, i.e., clear about her self-interest intention. As Khalil and Feltovich (2018) show in an experiment, people care about sincerity. People tend to punish, at their own cost, others who hide their intentions when they render a service.

Researchers have long recognized beneficence-as-informal-obligation (repayment) and indeed tend to restrict the term “reciprocity” to such beneficence. Sobel (2005) suggested the term “instrumental reciprocity”, whereas Boran (2006) suggested the term “principle of fairness” (see Cabral et al., 2014) to denote beneficence-as-informal-obligation. Indeed, Boran uses the term “principle of fairness” broadly enough to encompass all kinds of “moral obligation”—in contrast to formal (*ex ante*) agreements that act as the basis of “legal obligation.”

The idea of beneficence-as-informal-obligation probably informs many of the social norms urging beneficiaries to contribute to public goods or to eschew free-riding. Beneficence-as-repayment (moral obligation) is not limited to fairness regarding the use of public goods. It can also encompass the informal network of insurance—where members of a society help one in need while expecting this person to reciprocate and help others in a similar situation in the future. What sets such acts of moral (informal) obligations (beneficence-as-repayment) apart from formal obligations (justice) is the issue of *ex ante* agreement. While moral obligations do not involve *ex ante* contracts, formal obligations do.

This distinction permeates the three chapters that Smith (1976a, pp. 78–91) highlighted concerning the difference between what he calls “beneficence” and “justice.” He used the term “beneficence” in the strict sense to denote beneficence-as-informal-obligation, whereas “justice” in the same sense used above, i.e., beneficence-as-formal-obligation. (Smith also used the term “beneficence” to denote beneficence-as-altruism).

What is identified here as informal obligation, or the repayment of favors, also motivates Rabin’s (1993) modeling of fairness. Rabin uses the battle of the sexes game. In this game, both the husband and the wife benefit more by cooperating and going to the same event (e.g., boxing or the opera) than by the husband going alone to his preferred event (e.g., boxing) and the wife to her preferred event (e.g., the opera). It can be the case that the husband yields to the wife’s wishes and goes to the opera many times in a row. In this case, he feels cheated if she fails to reciprocate in accompanying him to the boxing event. He could retaliate by going to the boxing event alone, which would hurt him, in order to hurt her. In short, while the ultimatum game involves self-inflicting hurt in the context of *formal* obligation, the threat of retaliation in the battle of the sexes involves self-inflicting hurt in the context of *informal* obligation 

## Beneficence-as-Gift

Another act of beneficence that invites reciprocity is friendship-and-love—e.g., when the DM wishes a friend success in an endeavor, consoles a colleague for her loss, or simply expresses a smile with fondness when greeting a neighbor. These acts of affection need not be costly. However, the DM who expresses gestures of affection and kindness expects the others to reciprocate with similar gestures. It is constitutive of the nature of love that the recipient of love expresses the same affection in return.

Gestures of kindness, friendship, fondness, and love could be costly. The gestures could be accompanied by goods such as flowers, perfume, or cakes (Khalil, 2024a). The DM, who offers a gift to a friend on the occasion of his graduation from college, expects the friend to reciprocate with gestures of smiles and warmth. The reciprocation in the form of a gift, to be clear, is not a mandate, as it can be the case that the friend does not have a similar occasion or lacks the adequate budget. A gesture of kindness is sufficient. If the DM feels hurt about the reciprocated low-value gift, such feeling is not (or should not be) about the pecuniary (substantive) value--but rather about the symbolic meaning that for the DM did not correspond to her gift.

Furthermore, the reciprocated gift should not be of the exact same substantive value. Otherwise, it sends the wrong signal that the exchange is *quid pro quo*, i.e., as if the exchange fulfils a substantive informal obligation. Sobel (2005) calls beneficence-as-gift “intrinsic reciprocity”—expressly in contrast to the instrumental reciprocity that informs beneficence-as-informal-obligation.

Indeed, people across almost all cultures set solid walls, called “taboos,” to protect practices and rituals that they consider "gifts", i.e., relationships of friendship-and-love that are essential for the constitution of their respective communities (Steiner, 1999; Douglas, 2002). Taboos can be prohibitions to merely hold “unthinkable” thoughts deemed to undermine one’s group identity (see Fershtman et al., 2011). The taboos prohibiting thoughts aim at protecting what is sacred to group cohesion. Such taboos are usually associated with rituals, many of which reach the level of sacredness. The rituals can also be quasi-sacred as the case of holiday institutions that involve the exchange of gifts, which cements communal bonding and solidarity (Mauss, 1990; Khalil & Marciano, 2021a, 2021b). The solid walls ultimately protect the beneficence-as-gift rituals from being vitiated with self-interest pursuits.^5^

The solid wall that guards some gift exchanges—such as helping friends build a home—from *quid pro quo* exchanges may vary from one culture to another. Nonetheless, once the solid wall is defined for one culture, the people of this culture view actions that disregard the wall to be repugnant. For example, economists such as Roth (2007) are eager to set up markets that match sellers and buyers of human kidneys. However, these economists are frustrated by taboos that prohibit the commodification of human organs. These taboos are part of general taboos about the limits of market transactions (see Satz, 1995, 2008, 2012; Sandel, 2012, 2013), notwithstanding the call of many utilitarian thinkers to ignore such taboos (e.g., Posner, 1987; Becker & Elías, 2007; Ng, 2019; see Khalil, 2025b).

The debates surrounding repugnant exchanges and the limits of market transactions should, if anything, confirm that the motive for affection and love, on the one hand, and the motive for substantive benefit, on the other hand, are incommensurable. It is outside the scope of this essay to study the ultimate grounds of incommensurable preferences (Khalil, 2024c).

It is sufficient to point out, however, that the incommensurability of gifts and substantive gains does not warrant the postulate that friendship-and-love is completely autonomous from substantive gain (see Khalil, 2025b). As in the case of the feeling of integrity associated with the pursuit of justice, while this feeling may not be reducible to substantive gain, it is still related to substantive gain. Likewise, while the feeling associated with friendship-and-love may not be reducible to substantive gain, it is still connected to substantive gain, as shown in detail elsewhere (Khalil, 2024a).

## Beneficence-as-Allegiance

What is the nature of authority that a manager exercises vis-à-vis subordinates? Is such authority being a mirage, not different from the power that a customer exercises over a grocer? Two seminal papers in the economics literature—namely, Alchian and Demsetz (1972) and Jensen and Meckling (1976)—expressly argue that the firm–employee contract, where the authority relation is obvious, is no different from the customer–grocer contract, which also involves authority according to the authors of these papers. For these authors, the employment contract differs only in degree from the commercial contract, where one could involve more provisions and last longer than the other. The loyalty that a customer has toward the neighborhood grocer and vice versa, which can be the basis of authority, is no different from the loyalty that an employee has toward the employer and vice versa, which can also be the basis of same kind of authority.

It is true that the employment contract and the commercial contract, which can be between two or more firms, share some common features such as the institutions of justice, i.e., the reciprocal respect of the provisions of the contract. Such provisions—as a number of organizational economic theorists have argued, such as Coase, Williamson, and Winter (e.g., Williamson & Winter, 1993)—aim to protect the parties from expensive transaction costs arising from cheating occasioned by asymmetric information, from risk, and from lost opportunities to nurture shared competence, technologies, and skills. For these authors, whatever difference exists between the employment and commercial contracts is a matter of degree.

Masten (in Williamson & Winter, 1993) disagrees. It is true that a customer seems to “fire” her grocer when he starts to shop elsewhere—similar to the case when a firm “fires” an employee. However, for Masten, the firing in the former radically differs from the latter. Masten argues that the intra-firm contract, on the one hand, and the inter-firm (such as the customer-grocer) contract, on the other hand, differ in kind. The authorities that both types of contracts afford are not of the same genus.

Masten provides an analysis of corporate law to show that the firm and its contract with employees are not merely a more complicated market than the grocer and her contract with customers. The authority inside the firm, what this paper calls “allegiance,” may or may not involve asymmetric power. What matters is that such allegiance in intra-firm contracts differs from the asymmetric power that one customer, say a weighty customer, or one monopoly might have vis-à-vis relatively less powerful firms. The power of the manager, who expresses intra-firm contracts, enforces directives on the basis of the power of dismissal—which still differs from the power of a monopoly that is hegemonic, i.e., which imposes its provisions on the basis of its market-mediated asymmetric power of termination of relationships.

Asymmetric power in-itself—i.e., either the threat of dismissal of employees or the threat of termination of inter-firm alliances—cannot be the basis of why the employment contract differs from the commercial contract. As Masten tersely states,

If the power of management to enforce its directives rests solely on the threat of dismissal... then-a nominal duty to obey notwithstanding-the authority of management is no different from that of an independent transactor engaged in ordinary market exchange. Absent an express contract to the contrary, the ability to terminate negotiations and any further dealings is also the "only sanction" normally available to commercial transactors dissatisfied with the terms of trade currently tendered. Obviously, the claim of special authority in employment transactions cannot be based on the threat of termination alone (in Williamson & Winter, 1993, pp. 201–202).

Other papers confirm Masten’s affirmation (Khalil, 1995b, 1996a, 1997, 1998b). Specifically, neoclassical economic theorists of the firm—ranging from Coase, Winter, and Williamson—ultimately cannot offer a criterion that sets the institutions of power that underpin intra-firm contracts from the institutions of power that underpin inter-firm contracts. At best, these theorists offer descriptions of how the firm is a more command market than the free-wheeling bazaar (Coase), a bundle of “competence” (Winter), or a “nexus” of contracts (Willianson). These descriptions amount to a theory of institutions that does not differentiate the institutions underpinning market-mediated (inter-firm) contracts from the institutions underpinning firm-employee (intra-firm) contracts. That is, there is nothing special about the institutions that underpin organizations such as firms from institutions that underpin market-mediated, long-term alliances (see Khalil, 1995a).

What, then, is the ground upon which the employment/commercial contract distinction rests? How asymmetric power in intra-firm contracts, taking the form of allegiance, differs from asymmetric power in inter-firm contracts, which can even be cemented by what can be called “loyalty”. If the term “loyalty” is reserved to characterize the cement of intra-firm contracts, how does loyalty differ from “allegiance”—a term reserved to characterize the cement of inter-firm contracts? While both loyalty and allegiance involve trust (social capital), how does trust characterizing intra-firm exchange differ from trust that characterizes inter-firm exchange? (Khalil, 1993, 1994).

To understand the difference between the two kinds of trust or the allegiance/loyalty distinction, we must start with the master/servant relationship. Coase mentions this relationship in his 1937 classic paper (see Coase in Williamson & Winter, 1993). However, Coase basically fails to make such relationship the entry-point for understanding intra-firm contracts. To understand the origin of the master/servant relationship, it is useful to follow the thesis of Steiner (1949), a much neglected thesis in contemporary social theory other than occasional homage (e.g., Graeber & Wengrow, 2021, pp. 519–521; Khalil, 2025c).

On the basis of ethnographic studies and conceptual analysis, starting with Aristotle’s view of slavery, Steiner postulates that the master/servant relationship starts with *charity*, which is referred to here as “altruism.” A benefactor might decide to assist an orphan, a widower, or a disabled person. The beneficiary may, but not necessarily, express gratitude. The act of charity—without even the expression of gratitude—characterizes one type of cooperation, beneficence-as-altruism.

However, especially when the act of charity is repeated, the beneficiary may express, in addition to gratitude, submission-with-a-twist to the will and directives of the benefactor. Such submission involves the performance of labor tasks within the context of giving up some parts of one’s autonomy. That is, the labor tasks are not performed via the *quid pro quo* context, i.e., as commercial (customer-grocer) contracts. Rather, and here is the twist in submission-qua-allegiance, cooperation is grounded in giving up some parts of autonomy under the beneficiary’s “faith”—which is affirmed by the benefactor—that the benefactor is under the mandate of performing “guardianship”. While guardianship entails asymmetric power, it also entails the commitment to guard the best interest of the person (or nation) that is submitting to the will of the chieftain, employer, sovereign lord of the land, leader, or the state. This guardianship relation typifies the most obvious phenomenon: the parent‒child relationship.

Submission-with-a-twist, i.e., allegiance, differs from submission to a bully, warlord, mafia boss, or a hegemon foreign state. Submission to warlord/hegemon, as discussed below, does not involve the guardianship context. In contrast, allegiance is submission within the guardianship context. Thus, allegiance that characterizes the cooperative venture of employer and employee (or the state and subjects) is not of the same currency as the loyalty that can take place in *quid pro quo* transactions, such as customer‒grocer contracts or even more solid nexuses of contracts that characterize alliances of cooperative ventures.

The employer/employee, chieftain/subject, or state/citizen relationship can be fashioned along diverse ideological lines—including social contract ideology à la Hobbes and Locke or exploitation ideology à la Marx. Such ideologies deny, but for different reasons, that such relationships amount to allegiance understood as guardianship.

At its core, allegiance-qua-guardianship entails the giving up of some autonomy—which can be of different degrees of severity. The master/servant relationship captured, e.g., by Steiner, involves a severe degree of autonomy loss, whereas the modern employer/employee relationship regulated by the laws of the modern state involves moderate degrees of autonomy loss.

Disregarding the degree of severity, any asymmetric power wielded by the master or employer vis-à-vis the subordinate differs from the asymmetric power wielded by a monopolist in inter-firm (customer–grocer) contracts. While the power asymmetry of intra-firm contracts is couched in the faith expressed by the weaker party that the stronger party is acting as a guardian/parent, the power asymmetry of inter-firm contracts is, at first approximation, on the basis of relative bargaining power.

When the beneficiary submits to the will of the benefactor, the submission act transforms charity/altruism into a relation of submission-with-a-twist, i.e., allegiance. Such allegiance need not entail the loss of dignity—as it is the act of the beneficiary that initiates the relationship of altruistic help with the master/slave relationship.

It is outside the scope of this paper to show how allegiance, rather than loyalty, characterizes intra-firm contracts (see Khalil, 1996a, 1997, 1998b), not to mention political contracts between the ruler and the ruled (see Khalil, 2005, 2019, 2025c). However, it can be sufficient to start with Adam Smith’s critique of the social contract theory of the state (see Khalil, 1998a). Smith basically defaults Hobbes and Locke in conflating commercial contracts with political contracts, i.e., between the sovereign and the subject. In political contract, as postulated here regarding the intra-firm contract, the ruled expresses faith that the sovereign acts as a guardian. That is, the sovereign is mainly interested in the public interest of the organization, not in its own private interest (self-interest). For Smith, in addition to self-interest (which he calls the “principle of utility”), the political contract expresses allegiance insofar as it involves the “principle of authority”—i.e., what can be the term denoting submission-with-guardianship.

In unrelated text, Smith (1976a, pp. 50–66) identifies rank in society, where low-ranking people admire people of higher status, as the basic pillar of political order. A political community is stable when people at the bottom find the people at the top to be distinguishable and have traits worthy of admiration. Smith (1976a, pp. 50–52) traces such admiration to the quest of the DM to excel and achieve. For Smith, failing to have the desire to achieve is worse than death. The quest to achieve a goal usually leads the DM to admire the distinguished and powerful—although Smith complained that such admiration can be excessive, leading to what Smith calls disapprovingly “peculiar sympathy”. This sympathy is peculiar since it is not based on fellow-feeling with what the other fellow actually feels. It is based on fellow-feeling with how the other who occupies the high and distinguished station must feel and how I would feel if I imagined myself to share his station—what Smith characterizes as the “adulation” of the powerful in the sense of excessive admiration to the point of “obsequiousness” (Smith, 1976a, p. 52; see Khalil, 2002, 2005, 2019). Nonetheless, Smith continued to write that the quest to achieve, which entails rank in society, is the motor of the prosperity of the economic society.

Social rank expresses the acquiescence of the dispossessed and the poor to the authority of the rich and powerful. It takes the form of allegiance when it involves Smith’s principle of utility, i.e., the ability and willingness of the rich and powerful to employ the dispossessed and the poor. Such beneficence-as-allegiance expresses another form of cooperation/reciprocity—which actually typifies employer-employee relationships (see Khalil, 1996a, 1997, 1998b).

## Beneficence-as-Hegemony

Like cooperation based on allegiance, hegemony by definition involves power asymmetry. However, unlike cooperation based on allegiance, power asymmetry is based on bargaining power with respect to how to share the pie, where each party tries to reach the maximum that it can claim of the pie.

Two parties are involved in a relation of cooperation that is hegemonic insofar as one party accepts its own relatively weak bargaining power. That is, it accepts terms that enhance its wellbeing insofar as it is superior to the expected wellbeing earned if it resists the hegemon. Olson (1993, 2000) characterizes the cooperation between the state and the citizens as no different from the stationary bandit and the subjects that such bandit manages to subjugate, i.e., no different from the mafia and the residents of its neighborhood.. While the terrorized residents of the neighborhood submit to the directives of the mafia, this submission is without a twist, i.e., it does not involve a faith that the mafia is a guardian, i.e., an organization whose interest encompasses the wellbeing of the residents. The mafia is interested in the wellbeing of the residents, analogous to how a farmer is interested in the wellbeing of the chicken or cows of her farm—to extract the greatest benefit possible from the subjugated subjects while keeping them healthy for the following round of production.

According to Olson’s theory of cooperation, the state-qua-mafia is very careful not to overexploit the subjects, as the subjects then withhold investment. The withholding of investment results in a lower gross domestic product (GDP) than what an optimal taxation level would produce. That is, an excessive, suboptimal taxation level is a myopic policy, as it entails lower GDP and, consequently, lower public revenue in the longer term.

Inter-state geopolitical contracts, inter-firm alliances, monopolies, neighborhood-infested mafia, and governments operating in the aftermath of dysfunctional states are likely the apotheoses of cooperation on the basis of hegemony. Therefore, it seems difficult to characterize cooperation-as-hegemony as being benevolent. The expression “beneficence-as-hegemony” seems to be an oxymoron. The mafia organization or the government-acting-as-mafia does not act as guardian. Nonetheless, following Olson’s theory, the stationary bandit can be sincere about his intention, as the farmer is sincere vis-à-vis the chicken and cows: “I provide you with maximum support and nurture to ultimately benefit me—but it also optimizes your benefit as it is higher than the alternative option, i.e., expected utility in the wild where predators such as wolves and roaming bandits can make your life miserable and short.”

Market exchange among parties of equal market power, as in the case of the textbook model of perfect competition—or parties of asymmetric power but protected by a blind judiciary system (justice)—is indeed a special case of beneficence-as-hegemony. Under beneficence-as-hegemony, cooperation is beneficial if the hegemonic party is exclusively motivated by self-interest. Such interest is virtuous, i.e., beneficent, insofar as the hegemonic party is exclusively motivated by the promotion of self-benefit—ruling out deception/opportunism, self-aggrandizement, or malevolence.

The hegemon can actually be seen as a virtuous actor insofar as the outside option of the exploited is worse, namely, to be left in the wild to suffer. As argued elsewhere (Khalil, 2017c, 2017d), while the terms of the contract under beneficence-as-hegemony are exploitative, they do not necessarily entail injustice and, hence, do not necessarily entail deception or coercion that undermines beneficence. While the terms can be exploitative, the principles of justice do not apply insofar as the terms are not covered by an overarching roof of agreed-upon principles of justice that is supposed to govern both parties.

This approach addresses the issue of the *scope* of justice, which was mentioned above (see Khalil, 2025g). Briefly, major political philosophers, ranging from Plato, Locke, Kant, Marx to Rawls and Nozick, have offered great theories of how the ruler should relate to her subjects but without offering a theory of the coverage of such rules: Who should be considered members of the political community and, hence, protected by the ruler? and who should be consideredto fall outside the community and, hence, can be the legitimate subject of exploitation (see Khalil, 2017c, 2017d)? The failure to answer this question is behind the conflation of exploitation with injustice at the theoretical level. Aside from theoretical confusion arising from the inability to provide a theory of the scope of justice, we can still avoid confusion, guided only by de facto boundaries. Such boundaries allow us to characterize cooperation on the basis of hegemony as “exploitation” when one party takes advantage of another party who is weak—while such exploitation must be called differently, “injustice,” when both parties are members of a political community as defined by the scope of justice.

A contract can still be exploitative, i.e., operating under beneficence-as-hegemony, even when the terms of exploitation are tempered in light of altruistic considerations. A powerful partner in a cooperative venture may claim 70% rather than the 80% that she can claim out of altruistic preferences. Insofar as the division of the surplus is supposed to be 50–50 if the two parties are of symmetrical power, the cooperative exchange tempered with altruism is still hegemonic.

Exploitative cooperation under hegemony is beneficial not because of the altruism element. Even if there is no altruism element, such exploitative cooperation is beneficial insofar as the powerful party is motivated by self-interest—while the exploited party acquiesces for a simple reason: the offered exploitative relationship is better than the next best alternative option along the calculus of wellbeing.

## Beneficence-as-Grant

This paper defines grant-giving as acts of beneficence that express the amalgamation of two kinds of beneficence discussed above: beneficence-as-gifts and beneficence-as-altruism. Beneficence-as-grant signals love or what gifts usually convey while providing substantive help or what altruism affords.

Beneficence-as-grant is ubiquitous in modern economic societies (Boulding, 1973). Examples of beneficence-as-grant include financial help to a newly wed nephew, buying an automobile for one’s niece during her college years, or donating funds to one’s *alma mater* university. The grant does not only express the desire to help another person or organization. Otherwise, it would be beneficence-as-altruism. It also expresses feelings of affection, love, and bonding with the recipient.

The recipient of the beneficence-as-grant is usually expected to reciprocate with the expression of love, as in the case of beneficence-as-gift. The recipient is expected to show a similar affection to the uncle or the benefactor, who is helping with some sacrifice to the self. Furthermore, the recipient is expected to reciprocate and act as a benefactor in the far future if she becomes as successful as the original benefactor. Members of her social network expect her to share her wealth with her alma mater university, local hospital, or family members, as they supported her in the early stages of her career.

## Beneficence as Philanthropy

This paper defines philanthropy as an act of beneficence that expresses the amalgamation of two kinds of beneficence discussed above: beneficence-as-gifts and beneficence-as-*quid-pro-quo*. Beneficence-as-philanthropy signals love or what gifts usually deliver while providing substantive support for an expanded notion of self-interest.

Examples of beneficence-as-philanthropy are copious (e.g., Zunz, 2014). For example, a great and admired football player reciprocates the love of his fans by investing in a sport arena in a debilitatingly poor neighborhood—with the condition that it carries his name. Such a player not only expresses love but also helps expand the notion of the self. Hence, the act is an amalgamation of love and the pursuit of self-interest in an expanded sense. Given that the philanthropist’s ego is involved, the act is not beneficence-as-grant, as it is not about altruism.

Likewise, when an industrialist repays the love and support of her church through a hefty contribution to the restoration of the nearby library, the restored building carries her name. The industrialist not only expresses love but also provides substantive support for an expanded notion of the self. Again, such an act is about the glorification of one’s legacy and hence cannot be beneficence-as-grant.

The act of philanthropy can also take the form of volunteering to act as a guardian or a trustee of a corporation, university, or any organization. The guardian or trustee sacrifices current comfort to enhance the wellbeing of an expanded self. While the beneficiary reciprocates with love, the guardian enjoys such love by imagining how, once dead, the beneficiary would still express love toward her. With such an imagined link between the current self and the expanded self, the philanthropist manages to mitigate any pain endured by her divided psyche. The philanthropic act simply allows the current self to expand and, hence, to transcend the everyday pursuits of substantive satisfaction.

If we think that guardianship is one form of philanthropy, philanthropy is the primary motive behind the desire to raise, educate, and enable children. While this reason involves love, it also involves wanting to live beyond the grave, wanting to have a legacy that supports the interest of an expanded notion of the self. The desire to leave a legacy goes beyond the benefits that underpin investment/savings, namely, the intertemporal allocation discussed above. In beneficence-as-intertemporal-allocation, the DM’s duty is to provide what her parents provided. However, in beneficence-as-philanthropy, the DM provides beyond duty, namely, wants to leave an impact or legacy of an expanded notion of the self.

Thus, contrary to Becker’s (1976) theory of childrearing, it is not motivated by altruism. However, we must note that Becker viewed the altruistic act as an ego-centric act, i.e., as a mediated enhancement of pleasure derived from vicarious watching of the pleasure of the beneficiary (see Khalil, 2004). While the philanthropic act, such as raising children, has an element of ego-centrism, such an element is not à la Becker’s theory. In Becker’s theory, the ego-centric does not leave her biological skin. In the proposed view of beneficence-as-philanthropy, the supposed ego-centric expressly leaves the biological skin through the imagined expanded self.

Beneficence-as-philanthropy can also be argued to be the motive behind why people willingly and happily serve in the army to defend their tribe, their king, or their country. While this motive involves the love of country, it also entails an expanded notion of the self.

## Interdisciplinary Imperialism

This paper identifies ten types of cooperation/reciprocity, each informed by one or more of the five toolkits expressing respectively the five social science disciplines. While each social science is typified by one toolkit, how does it address types of cooperation/reciprocity that are not easily explained by its own toolkit? As stated at the outset of this paper, each social science discipline effectively invaded adjacent disciplines, sculpting the types of cooperation highlighted by such disciplines. This sculpturing, i.e., interdisciplinary imperialism, amounts to reducing the motive/preference highlighted by the adjacent disciplines as derived from one’s own toolkit. That is, each discipline essentially appoints itself as the “queen of the social sciences”—whereas the toolkits of the other disciplines are conceived as subsidiary to its own supposedly primary toolkit.

The stylized anthropologist—e.g., Douglas and even economists, such as Isherwood, who adopt the anthropological toolkit (see Douglas & Isherwood, 1996)—acknowledges the role of efficiency that is behind *quid pro quo*, intertemporal allocation, and possibly altruism. The stylized anthropologist also acknowledges the phenomena of power asymmetry, social roles, and divided-psyche as determinants of behavior. However, the stylized anthropologist exercises “anthropology imperialism” when he conceives of all these toolkits—and whatever corresponding cooperation/reciprocity types—as motives/preferences that cultural norms of the community under focus ultimately shape or mold to achieve the ultimate goal. The ultimate goal is the sustenance of the community, and hence, regardless of the motives/preferences that individuals possess, they are ultimately the products of the cultural norms of the community under study.

The stylized economist exercises “economics imperialism” when she conceives of all motives/preferences as the subject of efficient calculation. The economics imperialist is ready to recognize the quest after power asymmetry, the execution of tasks according to social roles to attain approval, the expression of cultural norms to experience love, and the consumption of goods to streamline the divided psyche. The economics imperialist, however, treats all these endeavors as reducible to a common metric—named “utility” or “welfare”—as if they are elements of a unidimensional objective function. The DM simply substitutes among these endeavors in light of relative scarcities to maximize the given objective function. As a result, all the diverse motives/preferences—which are captured differently by the different social science disciplines—are actually reducible to the single metric expressed by the unidimensional objective function. Consequently, if the judge insists on implementing the principles of justice, it is simply because he has not been bribed sufficiently, following the cliché that “every man has his price.” The DM, for the correct substantive compensation, is ready to sell principles of justice, bonding with community, dignity, and the quest after power.

The stylized political scientist acknowledges how efficiency, social roles, cultural norms, and divided-psyche determine behavior. However, the political scientist exercises “political science imperialism” when he analyzes all these motives as subsidiary to the ultimate goal: the attainment of power that is in excess of the power possessed by others. The ultimate goal of the DM is supposedly the sustenance of power asymmetry, where the quest after wealth, social acceptance, dignity, altruism, and philanthropy is to increase her power vis-à-vis the pertinent club of others.

The stylized sociologist recognizes that DMs seek power, value efficiency, live according to cultural norms, and undertake steps to amend the divided psyche. However, the stylized sociologist exercises “sociology imperialism” when she builds models of action where all these motives are subsumed to the ultimate end. Specifically, the DM is mostly interested in the attainment of social acceptance. Hence, when she seeks power, complies with cultural norms, puts under control the divided psyche, and acts according to efficiency, she actually fulfills her social role.

The stylized psychologist welcomes the diverse motives/preferences that motivate people to seek power, act efficiently, express fondness toward friends and family members, and act according to social expectations to receive approval from peers. However, the stylized psychologist exercises “psychology imperialism” when she conceives these actions as ultimately expressing the divided-psyche. For the stylized psychologist, the DM undertakes behaviors that are inconsistent because they express his incoherent and conflicting selves or undertakes behaviors as attempts to reconcile or pacify such selves (see Levine,^ 2016^).

## Three Primordial Satisfactions

The ten types of cooperation/reciprocity can be traced to three primordial genera of satisfaction: substantive, integrity, and transcendental. As detailed elsewhere (Khalil, 2025e), each genus actually encompasses many other kinds of action—which this paper ignores. This paper keeps its focus on the broad three genera:

1. *Substantive Satisfaction:* The DM enhances substantive satisfaction when one pursues self-interest or other-interest (altruism). Note that the pursuit of self-interest opens the door to enter into *quid pro quo* contracts, the most typical of which are market-mediated contracts. Regardless of whether they are market-mediated or bargaining-mediated, some of these contracts can amount to hegemonic cooperation. Additionally, the pursuit of self-interest disciplines the DM to respect the needs of the future self—i.e., undertaking optimal intertemporal allocation that entails avoiding indulgence in current consumption. Substantive satisfaction is equivalent to what the standard economist calls “utility,” “wellbeing,” or “welfare.”
2. *Integrity Satisfaction:* The DM nurtures integrity satisfaction when the DM sets up obligatory goals, i.e., contractual promises, and diligently pursues them optimally. This diligent pursuit involves abiding by the obligations that optimal decisions entail. This diligent pursuit gives rise to what the literature calls “warm glow”. Warm glow is a self-congratulatory, integrity feeling that arises when one completes a task and, hence, need not be restricted à la Andreoni (1989, 1990) to the performance of acts of altruism.
3. *Transcendental Satisfaction:* The DM augments transcendental satisfaction when she admires successful, high-status people, which can lead to allegiance. She also meets such satisfaction when she is engaged in gift giving, which fosters fondness and friendship with family members and loved ones. Such activities allow the DM to transcend her substantive everyday pursuits and, hence, permit her to desire goals and to adopt communities such as the family, the neighborhood, the temple, and the nation.

What about beneficence-as-grant and beneficence-as-philanthropy? They are also actions that satisfy transcendental goals—but more complex transcendental goals. Beneficence-as-grant is the amalgamation of beneficence-as-gift with beneficence-as-altruism: the grant giver not only wants to express fondness but also substantive support to enhance the wellbeing of the beneficiary.

In contrast, beneficence-as-philanthropy is the amalgamation of beneficence-as-gift with beneficence-as-*quid-pro-quo*. The philanthropist wants to leave a legacy, i.e., express the fondness and love of others through substantive support that actually expands his own sense of self. Such self-interest goes beyond the duty of enabling and equipping others as repayment of debt. The philanthropic act is an attempt to extend one’s life beyond the grave to empower loved ones to carry out their own vision.

The philanthropic act not only enhances the substantive satisfaction of the beneficiary but also enhances the beneficiary’s fondness toward the benefactor. The benefactor could be long dead, but the beneficiary still feels obligated to reciprocate and express love toward the long-gone philanthropist. For the philanthropist, she imagines while alive of such love beyond the grave and indeed acts to receive such love beyond the grave. Indeed, such imagined love expressed by the beneficiary confirms the proposed hypothesis: the philanthropist is motivated by self-interest, while the self here is expanded to include the benefit that beneficiaries in the far future will enjoy long after her death.

## Conclusion

This paper aims to clarify the different meanings of the common usage of the term “cooperation” and its corresponding term “reciprocity.” This paper identifies ten types of cooperation/reciprocity: i) *quid pro quo*; ii) intertemporal allocation; iii) altruism; iv) formal obligations (justice); v) informal obligations (repayment of favors); vi) gifts; vii) allegiance; viii) hegemony; ix) grants; and x) philanthropy.

Furthermore, this paper traces the ten types of cooperation to three primordial genera of satisfactions: substantive, integrity, and transcendental. Substantive satisfactions are met through the pursuit of self-interest, which may involve contracts that are hegemonic. The pursuit of self-interest also entails optimal intertemporal allocation as well as the abidance of the principles of justice vis-à-vis contemporaneous DMs. Altruism also enhances substantive satisfaction, which, in fact, is a sister-like motive of self-interest:

While formal and informal obligations are undertaken to satisfy interest (substantive satisfaction), the fulfillment of these obligations allows the individual to experience warm glow (integrity satisfaction).

Furthermore, the phenomena of gifts, allegiance, grants, and philanthropy allow the individual to transcend the daily pursuit of substantive aims and, hence, gain a sense of belonging and the feeling of aspiration to achieve aspired goals. Such transcendental sense and feeling allow the person to capture the meaning of her life.

Table 1 listed at the outset summarizes the ten-type taxonomy of cooperation/reciprocity, the three genera of satisfaction, and how each of the five social science disciplines and their toolkit expresses one or more types of cooperation/reciprocity.

Table 1 shows the proposed impartial platform, i.e., the transdisciplinary platform. There is no attempt to reduce what one discipline is most suited to discussing to a favored discipline/toolkit. There is equal recognition of all the toolkits. Table 1 shows where each toolkit shines and, simultaneously, becomes limited if it invades adjacent disciplines subjecting them to interdisciplinary imperialism. Table 1 provides a bird’s-eye view, helping researchers avoid interdisciplinary imperialism.

This paper shows how interdisciplinary imperialism is the necessary outcome of how five social science disciplines—namely, anthropology, economics, political science, sociology, and psychology—enforce discipline on their members. Each discipline effectively trains its members to abide by its postulated entry-point, i.e., an insular toolkit (mode of conception) of how to explain the plethora of human actions. Each insular toolkit is well suited to one type of motive/preference and, hence, can easily capture one type or two of cooperation/reciprocity. Moreover, when researchers within the discipline attempt to capture other types of motives/preferences and, corollary, types of cooperation/reciprocity, they effectively *sculpture* the other types after their preconceived toolkit. Such interdisciplinary imperialism can be avoided only if researchers within the discipline attempt to understand the other types of cooperation/reciprocity on their own terms.

However, if researchers step out of their respective discipline and attempt to understand the other types of cooperation/reciprocity according to how adjacent disciplines understand them, they would be undisciplined. They are usually shunned if not treated as outcasts by their colleagues within their own discipline—and for a few, they could become maverick thinkers in adjacent social science disciplines.

Starting with a transdisciplinary platform—i.e., one that acknowledges the different types of motives/preferences on their own terms—this paper was able to offer a ten-type taxonomy of cooperation and, with the exception of altruism, reciprocity. The taxonomy offers a balance between comprehensiveness of coverage, on the one hand, and parsimony of types of cooperation/reciprocity, on the other hand.

Future research may explore how different motives/preferences can intersect, functioning with different intensities in different societies. Furthermore, future research may focus on one society and record how the intersected motives can function with different intensities along the lifeline of the society. This would be an exciting research agenda.

## Data Availability

No datasets were generated or analysed during the current study.

## Appendix 1

### Implications of the Ten-Type Taxonomy of Cooperation/Reciprocity

#### Alternative Taxonomy

There have been many attempts to provide a comprehensive account of cooperation and sociality. This paper cannot review them. However, it is worth mentioning the attempt of Fehr and Gintis (2007). Similarly to this paper, they reject entry-points that postulate a primordial motive or preference—from which we can derive all other motives/preferences.

Fehr and Gintis, at first approximation, acknowledge the importance of two motives: self-interest that animates market transactions, on the one hand, and social approval that precipitates conformity to already existing social norms, on the other hand. For them, DMs may act according to self-interest in some situations but according to social norms on other occasions.

However, analytically speaking, one of the the two types of motives is derived from the other. For the individual to have a desire to please others and to adopt what others recommend, they can subsist in the long term if such recommendations assist the interest of the individual. That is, if such desire only promotes the interest of the collective at the expense of the individual’s interest, the individual pursues self-interest in a mediated way. Specifically, when the individual follows the expectations of others, she is pursuing her own interest *indirectly*, i.e., through the promotion of the interest of the collective. Thus, Fehr/Gintis’ two supposed motives amount to one motive: the individual in the market pursues self-interest in a direct way; the individual in society pursues self-interest in an indirect way. These proposed “two types” of preferences cannot stand symmetrical vis-à-vis each other.

#### Adam Smith on Justice and Beneficence

As mentioned above, Smith (1976a, pp. 78–91) dedicated three chapters to distinguish formal-obligation, which he called “justice”, from informal-obligation, which he dubbed “beneficence.” The thrust of the three chapters is to identify the new mores needed for a new socioeconomic order where commerce-based production and exchange takes on an independent sphere from other spheres of action. The new mores stress justice, i.e., formal (*ex ante*) agreements that inform legal obligations. As such, in these chapters, Smith highlights the foundation of the new order, where DMs can no longer appeal to reciprocity in the sense of moral (informal) obligation, i.e., beneficence-as-repayment.

As a corollary, many of the pre-commercial institutions that embody reciprocity in the sense of either beneficence-as-grant or beneficence-as-philanthropy must also be reshaped for the new modern order. As Smith states in *The Wealth of Nations*, in Chapter 2 at the outset, the root of the new set of institutions suited for commerce-based production and exchange is the pursuit of “own interest”, which calls for (formal and informal) obligations, some of which may turn into legal obligations. Smith went further and juxtaposed “own interest” against “the benevolence of the butcher, the brewer, or the baker” on the basis of “humanity” (see Khalil & Marciano, 2021b).

To be precise, Smith should have juxtaposed “own interest” against beneficence-as-altruism, on the one hand, and beneficence-as-love, on the other hand. In this manner, neither altruism nor love has vanished in the new modern order dominated by market transactions. Instead, people have relegated altruism and love to other spheres of the modern social order.

## Footnotes

1 The trolley problem illustrates the asymmetry of doing justice (do not harm) versus doing good (promote benefit). A bystander observes a trolley that is traveling down a path that will definitely kill five workers standing unwittingly on its path. Would the bystander intervene and pull down a level to divert the trolley to another path that will kill definitely one worker standing in an alternative path? Most people surveyed said they would save five over one unless the one is young, genetically related, or a romantic partner (see Bleske-Rechek et al., 2010*)*. However, it is not unreasonable to suppose that the surveyed people would be hesitant to save two over one. That is, people are ready to “let two die”, not intervene to do good, than to pull the lever and kill the one worker standing in the alternative path. This asymmetry—namely, between not-doing harm and doing good--seems to diminish as the ratio of saved to killed people rises.

2 There are other fields of scientific social inquiry, e.g., human geography and jurisprudence. However, this paper does not discuss them because each field basically lacks a mode of conception, an identifiable toolkit. Such fields amount to the amalgamation of different insights from the identified five social science disciplines.

3 While Boulding (1988, p. viii) used the term “economics imperialism,” Ralph William Souter earlier used the term denoting the same concept in his 1933 book, *Prolegomena to Relativity Economics* (see Backhouse & Medema, 2009).

4 Incidentally, Hume’s friend, Adam Smith (1976a) has proposed a theory of the sentiments where the sentiments are rooted in rational choice—or at least how a series of papers interprets Smith’s moral psychology (Khalil, 2010, 2017b, 2024c, 2025a).

5 There are other kinds of taboos, such as those that affirm deference to authority. Additionally, other kinds of taboos concern food prohibitions—such as the prohibition of eating cows, pigs, and other food. As Harris (1989) explains, food taboos generally arise from economic/environmental constraints. In this regard, taboos differ from disgust understood as behavioral reaction to suspected pathogens (e.g., Schaller & Park, 2011). While the set of taboos, as the term used here, protects exchanges based on beneficence-as-gift as opposed to substantive pursuits (e.g., self-interest), disgust aims to protect health that is part of the substantive pursuits.
